# Soft Biomimetic Underwater Vehicles: A Review of Actuation Mechanisms, Structure Designs and Underwater Applications

**DOI:** 10.3390/mi17020258

**Published:** 2026-02-16

**Authors:** Xuejing Liu, Jing Li, Yu Xing, Zhouqiang Zhang, Yong Cao, Yonghui Cao, Bo Li

**Affiliations:** 1 School of Mechanical and Electrical Engineering, Xi’an Polytechnic University, Xi’an 710048, China; lisa890329@163.com (X.L.);; 2 Unmanned Vehicle Innovation Center, Ningbo Institute of NPU, Ningbo 315103, China; cao_yong@nwpu.edu.cn; 3 School of Marine Science and Technology, Northwestern Polytechnical University, Xi’an 710072, China; 4 School of Mechanical Engineering, Xi’an Jiaotong University, Xi’an 710049, China

**Keywords:** soft biomimetic underwater vehicles (SBUVs), actuation mechanisms, biomimetic propulsion, electroactive polymers, underwater applications

## Abstract

The growing demand for marine resource development and in-depth exploration of the marine environment has positioned soft biomimetic underwater vehicles (SBUVs) as a research hotspot in the fields of underwater equipment and soft robotics. SBUVs are characterized by bodies made of flexible and extensible materials, integrating the dual advantages of softness and biomimetics. They can achieve muscle-like continuous deformation to efficiently absorb collision energy, while mimicking the propulsion mechanisms of marine organisms—such as fish and jellyfish—through undulating body movements or cavity contraction and relaxation. Such biomimetic propulsion is highly compatible with the flexible actuation of soft materials, enabling excellent environmental adaptability while maintaining favorable propulsion efficiency. Compared with traditional rigid underwater vehicles, SBUVs offer higher degrees of freedom, superior environmental adaptability, enhanced impact resistance and greater motion flexibility. This review systematically summarizes typical actuation methods for SBUVs—including fluid-powered actuation, shape memory alloy actuation, and electroactive polymer actuation—elaborating on their working principles, key technological advances, and representative application cases on SBUVs. These actuation mechanisms each offer distinct advantages. Fluid-powered systems are valued for high power density and precise motion control through direct fluidic force transmission. Shape memory alloys provide high force output and accurate positional recovery via controlled thermal phase changes. Meanwhile, electroactive polymers stand out for their rapid (often millisecond-scale) dynamic response, low hysteresis, and fine, muscle-like deformation under electrical stimuli. Current challenges are also analyzed, such as limited actuation efficiency, material durability issues, and system integration difficulties. Despite these constraints, SBUVs show broad application prospects in marine resource exploration, ecological monitoring, and underwater engineering operations. Future research should prioritize the development of novel materials, coordinated optimization of actuation and control systems, and breakthroughs in core technologies to accelerate the practical implementation and industrialization of SBUVs.

## 1. Introduction

Oceans cover approximately 71% of the Earth’s surface, harboring over 80% of the world’s biological resources, 50% of its oil and gas reserves, and abundant rare earth metals. However, human exploration of the deep sea (>200 m) currently remains less than 5%, while monitoring coverage of fragile ecosystems such as coral reefs stands at only around 12% [1]. This underscores the growing urgency to enhance marine resource development capabilities, strengthen marine ecological protection, and advance marine science and technology. Given the complex and dynamic marine environment, exploration and development activities are inherently reliant on advanced underwater equipment. In recent years, Autonomous Underwater Vehicles (AUVs), Remotely Operated Vehicles (ROVs), and underwater gliders have been widely deployed across tasks including marine resource prospecting, topographic mapping, subsea equipment maintenance, biodiversity surveys, and accident search and rescue. Nevertheless, most existing underwater vehicles comprise rigid components (e.g., motors, gears, bearings) paired with flexible skins. While offering advantages such as robust power output and high precision, they are plagued by inherent limitations including bulky structures, excessive noise, and constrained energy efficiency. These limitations have motivated the development of alternative approaches with different material compositions and design philosophies.

To systematically navigate the evolving landscape of underwater robotics and to precisely situate the biomimetic soft paradigm within it, a brief classification is beneficial. Underwater robots can be characterized along two primary dimensions: structural compliance and the degree of bio-inspiration. From a compliance perspective, they range from traditional rigid robots (e.g., conventional ROVs/AUVs), which offer precision and payload capacity at the cost of adaptability, to hybrid-stiffness robots that combine rigid frames with compliant elements, and finally to soft robots composed of elastomers and polymers that enable continuous deformation and safe interaction. Concurrently, their design inspiration spans from non-bioinspired geometric forms, to bioinspired morphology that replicates animal shapes often with rigid materials, and to biomimetic soft robots that emulate both the form and the compliant material properties of biological organisms.

This review concentrates on the intersection of the latter categories: soft robots employing a high degree of biomimicry. These vehicles, by mirroring the mechanical properties and actuation principles of aquatic life, present a compelling pathway to overcome the limitations of rigidity, promising enhanced agility, efficiency, and environmental compatibility in underwater operations. The following sections will provide a comprehensive analysis of the core aspects enabling this technology, including actuation, sensing, control, and fabrication.

Driven by advances in functional materials and additive manufacturing, biomimetic soft underwater vehicles (SBUVs) represent a transformative departure from rigid designs. Their bodies are primarily composed of compliant materials—such as elastomers, gels, and smart polymers (e.g., electroactive polymers)—with elastic moduli (typically ranging from ~10^4^ to 10^9^ Pa) that closely match those of biological tissues [2,3]. This intrinsic compliance addresses the key shortcomings of traditional robots by enabling safe interaction, seamless adaptation to unstructured environments, and the high-fidelity emulation of biological locomotion, such as the graceful undulation of fish or the pulsed propulsion of jellyfish. The key properties of the predominant soft material classes used in SBUVs, which directly determine their actuation compatibility and overall performance, are summarized in Table 1.

The diverse material properties outlined in Table 1 enable a variety of actuation mechanisms. The realization of these biomimetic capabilities fundamentally relies on novel actuation technologies that convert energy into muscle-like motion. Several distinct classes of actuation mechanisms have been developed: fluid-powered (pneumatic/hydraulic) systems generate high-force, dynamic motion by pressurizing embedded channels within elastomeric matrices [4]; electroactive polymers, including dielectric elastomers (DEs) for high-strain response and ionic polymer-metal composites (IPMCs) for low-voltage actuation, offer direct electromechanical coupling [5,6]; and shape memory alloys (SMAs) provide high-force, compact actuation through reversible phase transformations [7]. The fabrication of these complex, multi-material actuators is enabled by advanced techniques like soft lithography and embedded 3D printing, allowing for the precise embodiment of biological design principles [8].

Building upon this technological foundation, bionics provides the essential design blueprint. By investigating the structural and locomotor mechanisms of living organisms, bionics provides abundant inspiration for engineering design and robotics. Against this backdrop, soft biomimetic fish—an archetypal integration of bionics and soft robotics—has garnered increasing attention from scholars worldwide due to its unique flexible structure, agile swimming modes, and extensive application potential [9,10,11,12,13].

The development of soft biomimetic underwater vehicles dates back to the late 20th century, evolving from simple imitation to sophisticated design, and from theoretical exploration to practical implementation. Early iterations, which predominantly employed rigid skeletons with flexible skins and relied on mechanical or hydraulic actuation, achieved basic swimming but suffered from poor flexibility and limited concealment. A pivotal scientific advance was the in-depth analysis of propulsion modes—such as BCF (Body-Caudal Fin) [14], MPF (Median and Paired Fin) [15], and jellyfish pulsatile, which elucidated the intrinsic correlation among undulating waveform parameters (e.g., amplitude and wavelength), hydrodynamic characteristics (e.g., thrust coefficient and vortex intensity) and overall propulsion efficiency [16]. Concurrently, the advent of smart materials—notably the electroactive polymers mentioned above—revolutionized actuation. These materials provided muscle-like attributes—high energy density, intrinsic compliance, and fast response—directly addressing the limitations of early rigid systems. Together, this deepened hydrodynamic understanding and material innovation enabled soft vehicles to authentically replicate the muscle contraction-relaxation cycles of marine organisms, achieving highly biomimetic swimming performance.

In recent years, the integration of computational fluid dynamics (CFD) simulations, topology optimization methodologies, and deep reinforcement learning (DRL) algorithms has propelled research on soft biomimetic underwater vehicles into a new phase. From biomechanical and fluid dynamic perspectives, researchers have conducted in-depth analyses of the locomotor kinematics and fluid–structure interaction (FSI) mechanisms of various marine organisms, leading to the development of energy-efficient actuation strategies and adaptive control algorithms [17,18,19]. Concurrently, there has been a growing emphasis on enhancing the environmental perception and human–robot interaction (HRI) capabilities of SBUVs in unstructured underwater environments, aiming to achieve higher levels of intelligent and autonomous operation [20].

To date, significant advancements have been made in both the structural design and actuation technologies of SBUVs. On the structural front, researchers have developed a diverse range of biomimetic platforms with varied configurations and functionalities, enabling complex locomotor behaviors such as hovering, backward swimming, and rapid turning. On the actuation front, novel actuation technologies, including dielectric elastomers (DEAs), shape memory alloys (SMAs) and fluidic actuation have been successfully integrated into SBUV prototypes. These breakthroughs have continuously improved the flexibility, environmental adaptability, and functional expandability of SBUVs, thereby unlocking their application potential in scenarios such as deep-sea resource exploration, coral reef health assessment, and underwater infrastructure inspection.

## 2. Propulsion Modes of Marine Organisms

The exceptional locomotor capabilities of marine organisms, honed by millions of years of evolution, provide an invaluable blueprint for engineering efficient and agile underwater vehicles. This section first synthesizes the primary biological propulsion modes that inspire SBUV design, before elucidating the fundamental fluid–structure interaction physics that underpin and govern the performance of such bio-inspired systems.

### 2.1. Propulsion Modes: From Biology to Engineering Inspiration

**Figure 1:**
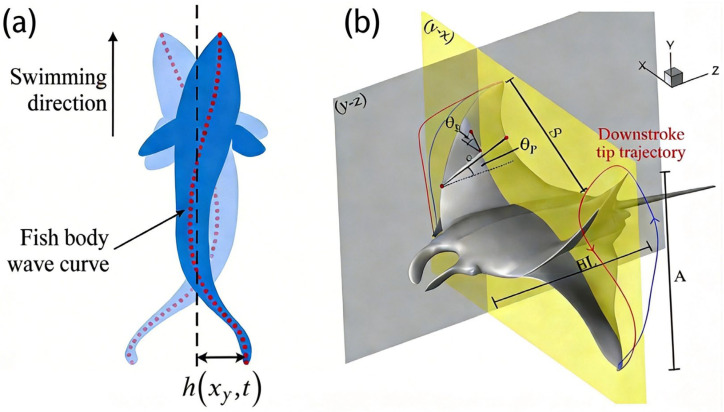
Schematic diagrams of BCF and MPF propulsion modes. (**a**) Schematic diagrams of BCF mode [14]. (**b**) Schematic diagrams of MPF mode [15].

Among underwater swimming organisms, Body–Caudal Fin (BCF) propulsion and Median and Paired Fin (MPF) propulsion stand out as the two most representative propulsion modes, shown in Figure 1, offering crucial references for the design of fish-like underwater vehicles.

Body–Caudal Fin (BCF) propulsion generates propulsive thrust mainly through longitudinal undulations of the body trunk or oscillations of the caudal fin. It includes four typical locomotor modes: (1) anguilliform swimming (exemplified by eels, with undulatory waves propagating along the entire body); (2) sub-carangiform swimming; (3) carangiform swimming; and (4) thunniform highspeed swimming. The last mode, seen in tuna, is dominated by oscillations of the caudal peduncle and caudal fin (core propulsive components). Theoretically, Lighthill’s slender body reaction theory has established a framework for elucidating the intrinsic correlation between body wave morphology (e.g., amplitude, wavelength), vibration frequency and propulsive efficiency [21]. Videler and Sfakiotakis’ comprehensive reviews have systematically categorized BCF modes of different fish and compared their propulsive efficiencies quantitatively [22,23]. Experimentally, Lauder’s work on mechanical visualization and flow field measurement has provided direct evidence for deciphering the caudal fin’s transient thrust mechanism and spatiotemporal evolution of its vortex structures [24]. Collectively, BCF propulsion has distinct advantages in high locomotor speed and superior efficiency. These traits make it suitable for fish engaging in long-distance rapid swimming, and highlight its potential as a biomimetic reference for high-performance underwater vehicle design.

In contrast, Median and Paired Fin (MPF) propulsion primarily relies on the movements of pectoral fins, dorsal fins, or anal fins to generate propulsive power and locomotor control. It encompasses three typical locomotor modes: (1) labriform swimming, which depends on pectoral fin flapping—common in coral reef fish and characterized by excellent maneuverability and hovering capability; (2) rajiform swimming (exemplified by rays and manta rays), which relies on undulations of wing-like pectoral fins to achieve lift and propulsion, making it suitable for low-speed precise operations; and (3) anguilliform or tetraodontiform swimming, which utilizes undulations of long dorsal fins or anal fins to expand the diversity of MPF propulsion modes [25]. Although MPF propulsion is inferior to the BCF mode in high-speed cruising performance, it exhibits prominent advantages in locomotor flexibility, low-speed stability, and small-scale spatial operations [26].

In addition, the pulsatile propulsion of jellyfish represents a unique paradigm. It achieves propulsion via bell contraction and relaxation, and utilizes vortex ring structures for passive energy recovery during the relaxation phase, thereby significantly enhancing energy utilization efficiency. Experiments by Gemmell et al. demonstrated that jellyfish can reduce active driving energy consumption through vortex persistence [27]. Furthermore, the Dabiri team revealed the vortex ring dynamics in jellyfish locomotion and its role in water transport, providing novel insights for the design of biomimetic propulsion systems [28].

To systematically translate these diverse biological strategies into practical engineering designs, a quantitative, metrics-driven framework is essential. The design rationale for distinct SBUV propulsion concepts is critically informed by quantitative hydrodynamic metrics and predictive models. The Large Amplitude Elongated Body Theory (LAEBT), established by Lighthill et al. in 1971, provides a foundational Lagrangian/reaction mechanics framework for modeling kinematics and estimating forces in fish-like locomotion [21]. Subsequent research has refined this theory and expanded the toolkit. Wang et al. (2010) demonstrated that active regulation of the Strouhal number (St) could enhance speed and efficiency in flapping tails [29]. Porez et al. (2014) developed a real-time, high-precision Newton-Euler model suitable for control applications [30]. Brouzet et al. (2025) further elucidated the dominant role of St in coupling vortex dynamics, thrust, and wake structure [31]. Consequently, contemporary SBUV design transcends morphological imitation by leveraging such universal dimensionless numbers (e.g., St, Reynolds number) as primary design parameters [31]. By targeting specific hydrodynamic metrics, designers can rationalize divergent SBUV concepts. For instance, BCF-type robots optimized for high-speed cruising might aim for a St at the lower end of the efficient range (e.g., 0.2–0.3) to maximize forward efficiency. In contrast, MPF-type robots designed for maneuverability and low-speed control might operate at a higher St (e.g., 0.3–0.4) to exploit enhanced vortex generation for thrust modulation. This metric-driven approach enables the systematic translation of biological principles into engineered functionality.

From the biomimetic design perspective, the BCF mode is well-suited for high-speed and high-efficiency propulsion, the MPF mode excels in maneuverability and hovering control, while jellyfish pulsatile propulsion provides an energy-efficient low-speed locomotion method.

The future research trend focuses on the integration and optimization of multiple propulsion modes, such as the coupled design of BCF and MPF, or the combination of jellyfish-like jet propulsion, to develop cross-scale, multi-mode and highly adaptable biomimetic propulsion systems [32,33].

### 2.2. The Pivotal Role of Fluid–Structure Interaction in SBUVs

The propulsion modes described in Section 2.1 represent more than kinematic archetypes to be copied; they are dynamic manifestations of a fundamental physical process: Fluid-Structure Interaction (FSI). For SBUVs, achieving true biomimicry—matching the efficiency, agility, and adaptability of marine life—requires moving beyond morphological imitation to actively engineer and exploit this two-way, nonlinear coupling between the compliant robot body and its fluid environment [34]. Unlike in rigid robots where FSI is often a parasitic effect to be minimized, in SBUVs, FSI is the central mechanism that governs energy transfer from actuator to thrust, determines stability and maneuverability, and ultimately defines the system’s performance envelope [35].

The theoretical foundation for understanding this coupling in aquatic propulsion was laid by Lighthill’s elongated body theory, which provided a seminal framework for modeling the reactive forces generated by an undulating body in an inviscid fluid [36,37]. This foundational work established the critical link between body kinematics and thrust generation, a principle that remains central to the analysis and design of BCF-type SBUVs.

At its core, FSI in SBUVs involves the fully coupled dynamics where inertial and elastic forces from a deforming structure directly influence the surrounding pressure and viscous fluid fields, which in turn dictate the structure’s subsequent motion [38]. This closed-loop interaction is what enables a fish’s flexible tail to generate a propulsive vortex jet. Pioneering experimental work by Lauder and colleagues, utilizing advanced flow visualization techniques like digital particle image velocimetry (DPIV), has been instrumental in deciphering these complex interactions and quantifying the underlying vortex dynamics. Their studies on live fish and robotic models have empirically detailed the three-dimensional vortex wake structures, the role of fin curvature, and the mechanics of thrust generation, providing indispensable validation and inspiration for engineering designs [39,40].

Therefore, the SBUV design problem inherently becomes a multi-physics co-design challenge: material properties, structural geometry, actuation strategy, and hydrodynamic performance cannot be optimized in isolation [41].

The choice of actuation technology dictates the dominant form of the FSI coupling, each presenting unique challenges. Smart Material Actuators (SMAs, EAPs) introduce strong material-field-fluid couplings. For instance, SMA-based systems face a thermo-fluid-solid interaction where the actuation frequency is critically limited by convective heat transfer to the surrounding water. Similarly, for Electroactive Polymers (EAPs) like DEAs and IPMCs, performance is governed by an electro-fluid-solid coupling, where the electric field-induced deformation must overcome fluid damping and added mass effects, which can drastically reduce effective strain and energy efficiency compared to in-air operation [38,41]. Fluid-Powered Actuators (Pneumatic/Hydraulic) embody FSI both internally and externally. The design challenge evolves into prescriptive waveform synthesis—precisely orchestrating the internal fluid pressure dynamics to generate an external body wave that constructively interferes with the flow for optimal thrust [35,42].

For pulsatile propulsion, inspired by jellyfish, the FSI dynamics revolve around vortex ring formation and control. Foundational research by Dabiri’s group has elucidated the critical role of vortex dynamics in jellyfish locomotion, demonstrating how the formation, pinching-off, and interaction of vortex rings govern swimming efficiency and maneuverability [43,44]. This body of work provides a physics-based blueprint for designing SBUVs that utilize unsteady fluid dynamics for propulsion and low-speed control [27].

Mastering these interactions necessitates sophisticated modeling. The field employs a structured hierarchy of approaches, from high-fidelity Computational Fluid Dynamics (CFD) coupled with Finite Element Analysis (FEA) for detailed analysis of vortex formation and force prediction [45], to reduced-order models (ROMs) vital for design iteration and real-time control [35,41]. These ROMs distill the essential physics—such as the resonance between structural natural frequencies and vortex shedding—enabling rapid performance prediction.

In summary, a deep understanding of FSI is not merely an add-on but establishes a mandatory co-design paradigm for SBUVs [34,41]. It mandates the synergistic integration of materials, mechanics, and hydrodynamics from the outset. The robot must be conceived as a unified system designed to engage in a specific, beneficial dialogue with its fluid environment, guided by principles established in both foundational biological studies and modern robotics research. This FSI-centric framework is critical for advancing the actuation, sensing, and control technologies discussed in the subsequent sections.

## 3. SBUVs Driven by Shape Memory Alloy Based Soft Actuators (SMASAs)

The working principle of Shape Memory Alloy-based Soft Actuators (SMASAs) stems from the reversible phase transition between austenite and martensite [7]. When stimulated by temperature or stress, the austenite phase transforms into martensite with twinned or needle-like characteristics. Upon removal of the stimulus, the crystal structure reverts to its original state, resulting in the “shape memory effect” and “superelasticity” [46,47]. In 1986, Tanaka proposed a one-dimensional thermodynamic model to describe this phenomenon [48]. Building on this, Brinson incorporated variable material parameters and internal martensitic variables to refine the constitutive model of SMAs [49]. Boyd and Lagoudas further advanced the field by integrating thermo-mechanical coupling and multi-dimensional dissipation mechanisms, facilitating the application of finite element simulation in SMASA engineering [50,51].

Although Ni-Ti-based SMASAs exhibit excellent performance in actuation and biomimetic applications, they still face limitations in high-temperature stability, recovery force, and cycle life. To address these issues, researchers have developed high-temperature SMASAs, such as NiTiHf alloys, by adding elements like Hf, Zr, and Cu to Ni-Ti. These modifications significantly broaden the phase transition temperature range and enhance high-temperature mechanical properties and cyclic stability [52,53]. Furthermore, performance degradation due to cyclic loading in corrosive marine environments is a critical challenge. To maintain transformation accuracy and functional lifetime under such conditions, material-based strategies have been developed. For instance, Hou et al. (2019) adopted laser powder directed energy deposition (LP-DED) technology to fabricate NiTi alloys [54]. The nanoscale Ni_3_Ti precipitates and interface mismatch formed inside the alloys can effectively reduce the phase transformation barrier and hysteresis, thereby significantly improving the fatigue life. In 2024, Liu et al. investigated laser cladded Fe-based SMA coatings [55]. This coating utilizes its own martensitic transformation to dissipate the impact energy generated by cavitation collapse, exhibiting excellent corrosion resistance and providing long-term protection for SMA systems in marine environments. Concurrently, from a system control perspective, the development of accurate self-sensing models offers a pathway to compensate for performance drift. As highlighted by Guo et al. (2024), the resistance sensing characteristics of SMASAs are fundamental for closed-loop control in deep-sea applications [56]. They developed a comprehensive model that captures the complex coupling between resistance, strain, stress, and temperature specifically for binary SMA deep-sea actuators under extreme conditions. By enabling real-time monitoring of the actuator’s state through resistance measurement, this model provides the theoretical foundation for implementing sensorless closed-loop drive control. Such a control strategy allows the system to perceive and adapt to performance changes (e.g., due to fatigue or environmental effects), thereby actively maintaining actuation accuracy and functional reliability over time, which is crucial for counteracting degradation in marine environments [56].

In terms of manufacturing, Kubášová et al. [57] reviewed the feasibility and challenges of additive manufacturing techniques, including Powder Bed Fusion-Laser Beam (PBF-LB) and Directed Energy Deposition (DED), for producing NiTi alloys. Prasad et al. demonstrated the potential of these methods in microstructure optimization but highlighted remaining issues such as compositional segregation, porosity, and residual stress [57,58,59].

Beyond conventional thermal actuation, researchers have also explored the magnetic field-driven properties of ferromagnetic SMASAs. Chernenko et al. investigated the controllable deformation capability of Heusler alloys such as Ni-Mn-Ga under magnetic fields, enabling magnetic actuation [60]. Heczko elucidated the underlying microscopic mechanisms through first-principles calculations and experiments [61]. Meanwhile, the use of SMASAs in micro/nano devices and medical implants imposes stricter requirements for fatigue life, surface condition, and biocompatibility. As early as 2014, Jani et al. emphasized the importance of microstructure and fatigue behavior in the design of superelastic stents [62]. Duerig’s and Mohd’s review highlighted the application potential of SMASAs in MEMS, implantable devices, and soft robots, noting that interfacial reliability and biocompatibility are key concerns [63,64,65]. Pelton systematically summarized the fatigue mechanisms of Nitinol, pointing out the significant influence of microstructure and cyclic loading on service life [66,67]. Consequently, researchers commonly employ methods such as surface treatment, composite structure design and microstructure control to improve the stability and durability of SMAs [68].

In addition, multi-scale numerical simulation has become an essential tool for addressing complex engineering challenges. Otsuka and Ren revealed the microstructural evolution mechanisms of SMASAs from a physical metallurgy perspective, while Bhattacharya introduced crystallographic models to analyze martensitic microstructures. In recent years, studies based on phase-field models, first-principles calculations, and multi-field constitutive relations have progressively enabled cross-scale design, spanning from the electronic level to functional device performance [49,50,69,70].

In terms of biomimetic applications, SMASAs have enabled diverse and innovative development pathways. In 2014, inspired by the hydraulic muscular structure of octopus arms, Cianchetti et al. developed a flexible robotic arm employing SMASA spring actuators, achieving multi-directional bending and telescoping motion underwater. This rigid-component-free design significantly reduced the risk of structural damage [71]. In 2018, drawing inspiration from black sea bass, Coral et al. embedded SMA wires into a continuous skeletal framework to mimic the motion of a fish spine. Experimental validation confirmed the biomimetic performance both in air and underwater [72].

The thermal relaxation time of SMASAs defines the physical limit of their cooling rate, thereby restricting the maximum actuation frequency. This bottleneck, stemming from limited heat dissipation, constrains the performance of SMASA-driven robotic systems. To overcome this, active thermal management strategies such as spray cooling have proven effective. Zhang (2024) demonstrated that a Spray-Cooled Antagonistic SMA Actuator (SCASA) could double the driving frequency of an SMA spring compared to forced air cooling, while also increasing driving frequency by approximately 40% [73]. The underlying principle is quantified by the thermomechanical coupling model proposed by Zhuo (2020) [74]. Their model introduces the dimensionless time-scale ratio λ (loading time vs. heat transfer time), which governs the thermal-mechanical response. Validated against experimental data, this model provides a predictive tool for designing SMA actuators under various operational conditions [74].

In 2020, Chen X. et al. [75] introduced the first untethered bionic fish driven by SMASAs, which realized the typical body-caudal fin (BCF) propulsion mode. By integrating an onboard power supply and remote control system, they overcame the limitations associated with earlier tethered actuation systems [75].

**Figure 2:**
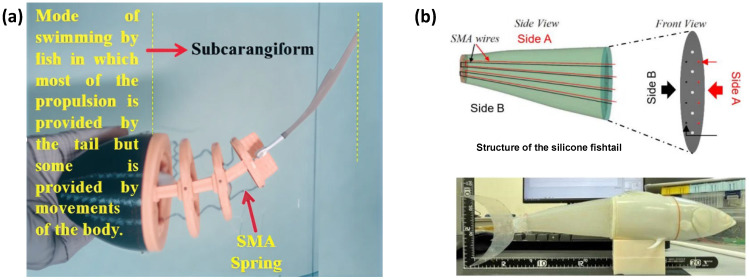
Examples of SBUVs driven by shape memory alloy based soft actuators (SMASAs). (**a**) Carangiform swimmer based on a SMASA spring mechanism [76]. (**b**) Wireless soft bionic fish with optimized SMASA parameters [77].

In 2021, Muralidharan M. et al. proposed a propulsion mechanism utilizing SMASA springs—a novel actuator derived from shape memory alloys (SMA) that exploits the material’s intrinsic pseudoelasticity [76]. Unlike traditional linear elastic actuators, SMASA springs exhibit distinctive nonlinear elastic characteristics, high damping performance, and precise deformation recovery. These properties enable them to accurately emulate the passive mechanical behavior of biological muscles during fish swimming. Integrated with a biomimetic, 3D-printed segmented trunk structure, this design successfully replicates the inter-segmental coordination characteristic of carangiform swimming kinematics—including the propagation of body wave amplitude and phase differences. This approach overcomes the motion asynchrony typical of conventional rigid-linked structures, allowing for a seamless reproduction of the natural undulatory motion of fish, as illustrated in Figure 2a.

Building upon this foundation, Ning K. et al. (2022) introduced two key advancements through computational fluid dynamics (CFD) analysis [77]. First, they optimized the fish body into a streamlined bio-inspired morphology that significantly reduces pressure drag by imitating the form of high-efficiency swimming fish. Second, they developed a synergistic actuation parameter system for the SMASA springs, carefully matching excitation frequency, pre-deformation, and cooling rate to enhance driving precision. By integrating these optimizations with a miniaturized wireless control module, the researchers created an untethered soft bionic fish (Figure 2b). A standout advantage of this platform is its high-precision maneuverability—exemplified by a turning radius of ≤1.2 body lengths and stable hovering capability in confined spaces. This represents a notable improvement over traditional wireless bionic fish, whose flexibility is often compromised by structural redundancy.

Expanding the scope of SMASA technology, Muralidharan et al. demonstrated in 2023, a jellyfish-inspired robot, where SMASAs drove flexible tentacles in pulsating motions. Equipped with integrated cameras and sensors, the system highlighted the fusion of cross-species biomimicry and real-time environmental sensing [78]. Most recently in 2025, Pan et al. [79] developed an untethered micro deep-sea robot based on SMASAs capable of multimodal locomotion by integrating legs and fins. It successfully performed swimming, gliding, shape morphing, and crawling operations at a depth of 10,600 m in the Mariana Trench, offering valuable design insights for the next generation of miniature deep-sea actuators and SBUVs [79].

In the field of underwater robotics, shape memory alloy (SMA) has established a mature bionic application system leveraging its flexible actuation advantages, exhibiting a multi-dimensional upgrading trend. Its applications have expanded from single-species simulation to cross-species multimodal bionics, yielding various flexible configurations. Technically, it has achieved breakthroughs in untethered wireless control and integrated sensing functions to form an integrated solution. Application scenarios have extended from conventional underwater environments to the 10,000 m deep sea, enabling robots to perform multi-mode combined motions with broad application prospects [79].

## 4. SBUVs Driven by Fluid-Powered Soft Actuators

Fluid-powered soft actuators represent a class of actuation systems that generate mechanical motion by regulating fluid pressure or flow, thereby converting fluid energy into mechanical work. In soft biomimetic underwater vehicles (SBUVs) designed to emulate fish-like locomotion, these actuators typically produce body undulation or bending through the dynamic control of fluid-filled chambers or embedded channels. The technology offers inherent advantages such as structural simplicity, rapid dynamic response, and high motion control precision. However, it also faces notable limitations, including dependence on an external fluid supply system—which increases overall complexity and energy consumption—as well as performance constraints arising from the physical properties of the driving media (e.g., compressibility of gases, viscosity of liquids) and the mechanical behavior of soft materials. These factors collectively influence key metrics such as swimming speed, maneuverability, and environmental adaptability [80,81,82,83]. Based on the working medium employed, fluid-powered soft actuators are broadly classified into two main categories: those driven by pneumatic systems and those driven by hydraulic systems, which will be elaborated in the following sections.

However, for SBUVs requiring effective thrust, the discussion must extend beyond mere classification to consider how these fluid characteristics impact the temporal fidelity of body undulations. Achieving high-fidelity reproduction of body undulations for effective thrust demands a deeper understanding of how fluid characteristics govern the temporal dynamics of waveform generation. First, the compressibility of the fluid fundamentally determines the system’s stiffness and response bandwidth. Hydraulic systems, utilizing nearly incompressible liquids, offer significantly higher stiffness and faster force transmission compared to their pneumatic counterparts, enabling more precise tracking of time-varying pressure commands and thus more accurate waveform synthesis [84]. Second, the viscosity of the working fluid, often considered a source of energy loss, can be strategically harnessed. By designing internal networks of slender fluidic channels, the viscous flow resistance can be leveraged to create deliberate, time-dependent pressure gradients between interconnected chambers. This allows a single pressure input to generate complex, sequential bending motions—effectively programming the undulation waveform through fluidic design itself [85]. Finally, accurate dynamic modeling that captures the nonlinear interaction between the fluid, the soft structure, and the external water is crucial for predicting and controlling the transient response. For instance, models incorporating the viscoelasticity of the actuator material have been developed to describe the dynamic bending behavior of hydraulic actuators, providing a foundation for model-based control strategies to enhance waveform precision [86]. Therefore, the pursuit of high-speed and maneuverable SBUVs necessitates a co-design philosophy that synergistically considers the selection of the working fluid, the morphological design of the fluidic circuit, and advanced control algorithms that account for these fluid-mediated dynamics.

### 4.1. SBUVs Driven by Pneumatic Soft Actuators

Considerable attention has been focused on pneumatically driven soft biomimetic underwater vehicles (SBUVs), particularly in pisciform configurations, as they represent an innovative platform for underwater propulsion. Their actuation mechanism relies on the expansion of internal cavities under pressurized air. A foundational advancement in fabricating such actuators is the “bubble casting” technique introduced by Jones et al. [87], which enables the scalable and precise manufacturing of soft actuators with complex, embedded fluidic channels, thereby providing a versatile material platform for SBUVs. Focusing on the actuation principle, the static modeling framework developed by Sun et al. [88] quantitatively elucidates this pressure-to-motion conversion process, explicitly linking fiber-reinforcement patterns and internal pressure to the bending behavior and block force of the actuators, which forms a crucial theoretical basis for designing asymmetric structures with predictable deformation outcomes. A representative implementation of these principles incorporates multiple pneumatically driven soft actuators arranged along the body or tail. When pressurized in a coordinated sequence, these actuators can generate traveling wave deformations or targeted bending to produce propulsion and enable steering maneuvers, as demonstrated in various robotic fish platforms [89].

The performance of pneumatic soft actuators is highly dependent on engineered anisotropy. The underlying principle is the active regulation of local strain energy through non-uniform stiffness distributions—creating compliant zones for bending and stiffer regions for shape maintenance. This guides deformation precisely, suppressing buckling and improving accuracy. Microstructural patterning refines this to a mesoscopic scale; anisotropic architectures (e.g., lattices) elicit direction-specific mechanical responses. This is demonstrated by pattern-forming pneumatic metamaterials: a periodic hole array forms a tunable lattice where distinct pressure patterns selectively activate microscopic buckling modes, leading to precise, reversible shifts in macroscopic stiffness and deformation [90]. For direct shape control, kirigami-inspired designs use patterned cuts as programmable pneumatic hinges. Independent pressure regulation in each hinge chamber controls local stiffness and deflection, enabling active, precise programming of complex curvatures from a flat sheet [91]. These principles are implemented via advanced fabrication. Zhang et al. (2019) developed a DLP 3D printing process for customizing micro actuators, enabling diverse, precise deformation modes [92]. Wang et al. (2024) introduced a volume-preserving cutting and bonding (VPCB) concept with an inverse design framework, offering a novel pathway for reusable actuators that ensure versatility and precise control [93].

The main structure of pneumatic SBUVs is typically fabricated from elastomeric polymers such as silicone rubber and polyurethane [94]. These materials are selected not only for their high flexibility and elasticity—enabling them to closely mimic the mechanical properties of biological fish tissue—but also for their ability to undergo reversible deformation in response to variations in gas pressure. This behavior provides the essential structural basis for biomimetic locomotion. For instance, a body segment made of silicone rubber can achieve large-amplitude bending and stretching during cyclic inflation and deflation, accurately reproducing the body undulation observed in live fish during swimming.

In terms of actuation structure, elastomeric chamber-based pneumatic soft actuators represent the current mainstream technical approach. These actuators generate biomimetic motion through spatially asymmetric distribution of materials or elastic modulus and are predominantly employed in tail-driven SBUV configurations. Early straight cylindrical elastomeric chambers, while structurally simple, were prone to radial expansion under high pressure, compromising motion stability. To overcome this limitation, Yoel Shapiro et al. introduced a double bellows actuator with a single degree of freedom in 2011, capable of achieving bending angles exceeding 180° under moderate pressure with excellent performance [95]. In 2014, Correll et al. further enhanced this design by embedding rib structures inside the cavity, decomposing the overall large deformation into localized deformations of multiple small air chambers. This modification significantly improved the actuator’s motion accuracy and reliability [96].

In the area of modeling and control, continuous efforts have been made to improve the predictability and performance of these systems. In 2018, Zhou W et al. conducted modeling and analysis of symmetric-chamber soft actuators for multi-chamber pneumatic drive structures [97]. Two years later, Guo Di et al. improved mechanical output through parametric optimization of chamber layout and geometry, validating their approach via finite element analysis and experimental tests [98]. More recently in 2023, Pushpendra Kumar et al. proposed a refined modeling method incorporating linear correction terms for soft pneumatic actuators (SPAs), effectively reducing the discrepancy between analytical models and finite element results, thereby advancing the understanding of material nonlinearity in soft structures [99].

In terms of system implementation, Marchese et al. [89] achieved a milestone in 2014 by developing the first untethered pneumatic soft robotic SBUV. Powered by an integrated high-pressure gas cylinder, the robot generated alternating left-right tail oscillations, establishing an important reference for subsequent untethered SBUV designs [89]. In 2022, inspired by the red muscle architecture of biological fish, Liu et al. [100] designed a biomimetic tuna prototype incorporating a pneumatic silicone structure. By embedding fluidic actuation units as artificial “muscle fibers” within a flexible matrix, they enabled tail fin undulation through alternating pressure excitation. This approach improved payload capacity while preserving body flexibility and motion smoothness [100].

**Figure 3:**
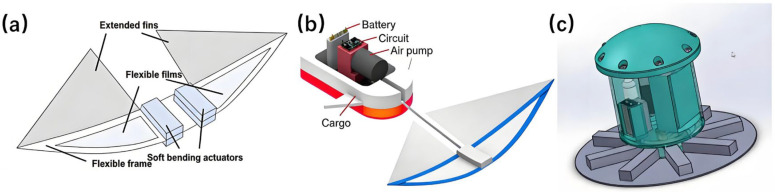
Examples of SBUVs driven by pneumatic soft actuators. (**a**) Lightweight swimmer with butterfly stroke propulsion [101]. (**b**) Manta ray-inspired soft swimmer [102]. (**c**) Tethered jellyfish robot with SPC actuators [105].

The application of pneumatic soft actuators in SBUVs has led to diverse and innovative platform designs. Figure 3 provides visual examples of several representative prototypes that highlight distinct design strategies. To achieve high-speed and efficient propulsion, Chi et al. (2022) developed a lightweight soft swimmer that employs a bistable snapping mechanism within its body [101]. This mechanism, based on flexible pneumatic bending modules and pre-curved airfoils, utilizes a hairpin-like structural configuration to constrain motion between two stable states, thereby ensuring high actuation accuracy and repeatability. The resulting robotic platform, which performs a butterfly stroke-like motion, is shown in Figure 3a [101]. Building on this concept, Qing et al. introduced a monostable bionic vehicle in 2024 (Figure 3b), which achieves periodic flapping using only a single drive input. This design achieves motion speeds twice that of the bistable structure while simplifying control logic [102].

A key challenge, however, lies in reconciling these models [97,98,99] with real-world underwater tests, where SBUV deformation is complicated by turbulence, viscoelastic relaxation, and asymmetric loading. To bridge this gap, research is converging on two complementary strategies. First, the development of high-fidelity, coupled fluid–structure interaction models—which explicitly incorporate material nonlinearity and transient flows—provides a more realistic, albeit computationally intensive, simulation framework [103]. Second, there is a growing emphasis on data-driven model updating and adaptive control, whereby simplified real-time models are continuously calibrated using sensor feedback (e.g., pressure, curvature, IMU data) to compensate for unmodeled disturbances such as turbulence and material drift, thereby maintaining robust performance in complex environments [104]. Together, these strategies furnish a crucial framework for addressing the core challenges facing SBUVs. Successfully translating these advanced modeling and control paradigms to the marine environment—by fully accounting for the unique conditions of hydraulic actuation, biofouling, pressure effects, and soft material behaviors—will be pivotal in evolving pneumatic SBUVs from laboratory prototypes into robust, deployable systems, ultimately closing the gap between predictive simulation and reliable underwater operation.

The scope of pneumatic soft actuation has expanded beyond fish-like platforms to include other aquatic organisms such as starfish and octopuses. In 2011, Shepherd et al. developed a multi-gait quadrupedal soft robot using soft lithography, replicating locomotion patterns of starfish and octopuses [106]. The following year, Morin et al. incorporated microfluidic networks to enable functions such as color and temperature variation, further mimicking cephalopod camouflage mechanisms [107]. In 2019, Joshi A et al. [105] created a tethered biomimetic jellyfish robot (Figure 3c) based on an inflatable Soft Pneumatic Composite (SPC). Operating at 70 psi, it demonstrated a payload capacity of 100 g and an ascent speed of 160 mm/s, highlighting its potential for loaded operation [105]. In 2022, Bell et al. developed the autonomous starfish-inspired robot ASTER-bot, which utilizes five bidirectional bellows actuators with a 270° motion range to navigate complex environments [108].

In summary, research on pneumatic SBUVs has progressively overcome limitations in motion precision, endurance, and environmental adaptability through structural innovations—such as red muscle-mimicking designs and multi-stable mechanisms—functional expansions including untethered operation, and cross-species biomimetic strategies. Future advances in soft material performance, multimodal control algorithms, and interdisciplinary integration with microfluidics and smart materials are expected to further promote the application of pneumatic soft robots in underwater exploration, biological interaction studies, and environmental monitoring.

### 4.2. SBUVs Driven by Hydraulic Soft Actuators (HSAs)

Soft biomimetic underwater vehicles (SBUVs) driven by hydraulic soft actuators (HSAs) operate on a principle similar to their pneumatic counterparts, generating motion by controlling the injection and release of liquid into flexible internal chambers to induce periodic expansion and contraction. The fundamental working principle enabling this motion lies in the direct conversion of fluid pressure into mechanical deformation. When incompressible hydraulic fluid is pumped into the sealed, compliant chambers of the actuator, the internal pressure rises. Due to the inherent elasticity of the actuator’s material (e.g., silicone rubber) and, crucially, by design with a structural asymmetry, the chamber expands non-uniformly [109]. This controlled, directional expansion translates macroscopically into bending, twisting, or extending motions of the actuator body. By coordinating the pressurization sequence and magnitude across multiple independent chambers through valves, programmed deformation patterns—such as the undulatory body wave of a fish—can be synthesized. In essence, it transforms the hydraulic energy from the pump into precise shape-changing kinematics of the soft structure, thereby generating propulsion.

The advantages of hydraulically driven SBUVs stem from the synergy between the inherent compliance of soft materials and the precise force control enabled by hydraulic systems. Compared to pneumatic actuation, the incompressible nature of the working fluid delivers higher force density and response bandwidth, enabling stronger and faster actuation critical for mimicking the burst acceleration and sudden braking of fish [110,111]. Relative to traditional rigid motor-propeller propulsion systems, its strengths lie in inherently low noise and vibration characteristics. Since the primary noise source stems from low-frequency flow pulsations rather than cavitation or gear meshing, its acoustic signature is distinctly different, with a substantial reduction in sound pressure level within key frequency bands. This grants it irreplaceable value for underwater observation and biological studies that impose stringent acoustic stealth requirements [112]. However, attaining these advantages comes at the cost of increased system complexity, including challenges such as high-pressure sealing, miniaturization and integration of pump-valve systems, and potential environmental leakage of the working fluid. These issues constitute the major engineering challenges for this technological pathway. Beyond stealth, the dynamic performance and energy efficiency of the hydraulic system are also paramount for sustained and agile locomotion, drawing attention to phenomena such as resonant actuation.

Resonant states induced by hydraulic actuation have a significant impact on locomotion performance. Konishi et al. proposed a scheme aimed at achieving high-power output from a hydraulic reciprocating pump driven by multi-layer piezoelectric elements (MLPE) [113]. The scheme is summarized as follows: (i) reducing the diaphragm spring constant to enable MLPE elongation; (ii) applying static pressure to the working fluid in the hydraulic circuit; (iii) connecting the pump valves and pressure chamber to piping with the same resonant frequency. By adopting this scheme, the maximum power and efficiency of the working pump were increased to 62 watts and 35%, respectively, which can be utilized to enhance the movements and performance of biomimetic fish in the future. This case confirms that deliberately exploiting hydraulic resonance is a proven efficiency-boosting strategy. Extending this principle to SBUVs, the core idea is to match the natural frequency of the fluidic circuit with the desired undulation frequency of the soft actuator. In resonance, the system requires minimal input energy to sustain large-amplitude fluid oscillations, meaning the same actuation pressure can generate body undulations with greater amplitude or higher frequency—both key for increasing thrust—while reducing onboard power consumption. Therefore, future work could focus on co-designing the soft actuator’s mechanical properties and the hydraulic drive system’s parameters to deliberately exploit such resonant responses, thereby optimizing energy transfer from the pump to the propulsive waveform.

The performance of SBUVs driven by hydraulic FPSAs largely depends on the efficiency of the hydraulic system. Katzschmann et al. systematically evaluated six hydraulic actuation configurations in 2016, identifying that a system integrating a brushless centrifugal pump with a rotary valve achieved the highest operational efficiency within the tested frequency range [114]. In 2022, Haji and colleagues performed nonlinear dynamic modeling of a Fish Tail Fluid Actuator (FTFA), constructing a mathematical representation approximating a continuous soft system via the assumed mode method. Their simulations indicated that multi-order resonance states can substantially enhance swimming efficiency [115]. To further improve modeling accuracy, Mahdi Bamdad et al. introduced a hybrid analytical–finite element approach in 2023. By incorporating factors such as cross-sectional geometry and number of fluid channels, this method effectively improved both deformation prediction accuracy and computational efficiency for soft hydraulic actuators [116].

The realization of such high-performance, resonant-capable systems hinges on the foundational design and fabrication of the soft actuators themselves. Fiber reinforcement and fabric composite technologies serve as the core enablers for enhancing the performance of soft actuators and soft robotic manipulators. Relevant research spans multiple dimensions including material optimization, modeling methods, control strategies, and system integration. In studies on hydraulically driven fiber-reinforced elastomer actuators, researchers compared polyurethane (PUR), thermoplastic vulcanizate (TPE), natural rubber (NR), and polydimethylsiloxane (PDMS), determining that PDMS combined with aramid fiber wound at 240 turns per 170 mm length represents the optimal configuration, offering the best pressure resistance and force output performance [117]. To address the inherent strength limitations of soft materials, high-strength hydraulic soft manipulators based on anisotropic fabric composites further expand the application boundaries. Using knitted aramid fabric (providing stiffness) and elastic fabric (enhancing range of motion), two types of actuators, AFA and EFA, were fabricated. These were integrated to form an elephant-trunk-like multi-degree-of-freedom system with a three-fingered fiber-reinforced soft gripper. The system can operate at a driving pressure of up to 1.5 MPa with a maximum payload of 1483 g. It demonstrates excellent performance across metrics such as a maximum bending angle of 121° for EFM, a maximum output torque of 11.2 N·m for AFM, and a stable response time of 1.08 s for AFA. This enables it to adapt to grasping and heavy-load support tasks involving objects of varying shapes and stiffness [118]. In actuator design and fabrication, Sangian et al. [119] developed a compact hydraulic actuator in 2015 capable of a 23% stroke and generating 26 N of isometric force within approximately one second. Their work also systematically analyzed the influence of geometric and material parameters on actuator performance [119]. That same year, Marchese et al. [120] investigated three types of silicone-based hydraulic actuator structures—ribbed, cylindrical, and corrugated—along with corresponding fabrication techniques, including lamination, telescoping pin molding, and lost-wax casting. This study provided a foundational manufacturing framework for soft actuators with complex internal channel architectures [120].

Although pneumatic actuation remains dominant in SBUV research due to its simplicity and controllability, hydraulic actuation continues to hold significant value in specialized applications. In 2015, Katzschmann et al. developed an autonomous hydraulic driven soft robotic fish using a novel closed-loop system with water as the working fluid [121]. This robot achieved caudal fin propulsion and yaw motion through internal channel control and introduced a soft manufacturing process capable of fabricating arbitrary flow paths. To enhance motion flexibility, the same team introduced “Sofi,” a soft robotic fish with 3D maneuvering capability, in 2018 [122]. This untethered prototype could operate for up to 40 min with an average speed of 0.51 BL/s, demonstrating its potential as a tool for seabed exploration and marine biological observation.

In the domain of energy systems, Aubin et al. proposed in 2019 the use of hydraulic fluid as both an actuation medium and an energy carrier [123]. Integrated into a lionfish-inspired robot, a synthetic vascular system with high energy density enabled a theoretical maximum operating time of 36.7 h, paving the way for integrated energy–actuation design in soft robotics.

Regarding motion performance, Ju I. et al. developed a soft robotic fish in 2023 featuring a Hydraulic Variable Stiffness (HVS) mechanism [124]. By actively modulating tail stiffness, they achieved a 118% increase in average swimming speed and reduced the turning radius to nearly one-quarter of the original. Also in 2023, Xia et al. applied origami techniques to tail design, effectively suppressing radial expansion and improving energy efficiency by 92.3% compared to conventional tail designs [125].

Liu et al. [126] successively developed two generations of hydraulic soft robotic tuna in 2023 and 2025. The first, HasorTuna, employed McKibben artificial muscles as driving units to realize high-frequency and efficient swimming [126]. The more recent HyperTuna adopted a four-pump coordination strategy and dual-servo independent control, substantially improving tail-beat precision and motion agility [127].

**Figure 4:**
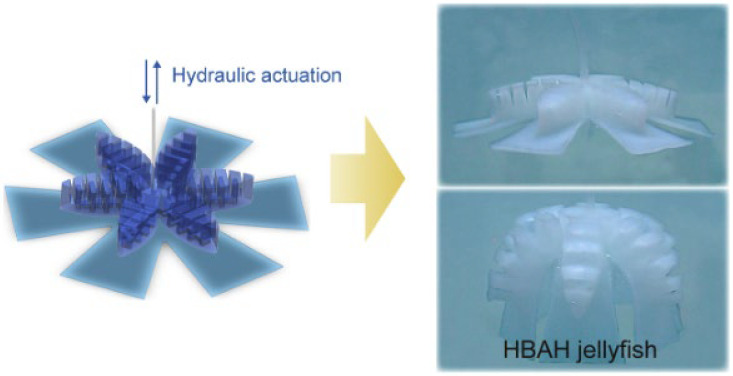
Example of SBUV driven by hydraulic soft actuators. Bioinspired hydrogel jellyfish with mechanical flexibility and acoustic transparency [128].

Hydraulic actuation has also been extended to the biomimicry of other aquatic organisms. In 2022, Zhang et al. created a hydraulically driven bio-inspired hydrogel jellyfish (Figure 4), which utilized hydraulic actuation to achieve large deformation while maintaining acoustic transparency, highlighting its potential for adaptive underwater applications [128].

In summary, through continuous innovations in system architecture, stiffness modulation, structural design and energy integration, hydraulically driven SBUVs have progressively overcome limitations in motion performance and energy efficiency. Future advances in multi-actuator coordination and material–structure–function integration are expected to further unlock their application potential in areas such as underwater exploration and ecological monitoring.

## 5. SBUVs Driven by Electroactive Polymers (EAPs)

**Figure 5:**
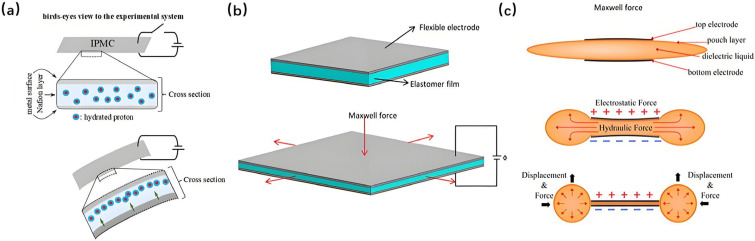
Schematic Diagrams of Three Types of EAPs (**a**) Schematic Diagram of the Working Principle of Ionic Polymer–Metal Composites (IPMCs) [129]. (**b**) Schematic Diagram of the Working Principle of Dielectric Elastomers (DEs) [130]. (**c**) Schematic Diagram of the Working Principle of Hydraulically Amplified Self-Healing Electrostatic (HASEL) Actuators [131].

Electroactive polymers (EAPs) have emerged as a pivotal class of actuation materials for soft biomimetic underwater vehicles (SBUVs), enabling highly adaptive, low-noise, and biologically plausible locomotion in aquatic environments. Their intrinsic compliance, efficient energy conversion, and scalability make them particularly suitable for mimicking the undulatory and fin-based propulsion mechanisms observed in marine organisms. Among the most widely studied EAPs for underwater applications, ionic polymer–metal composites (IPMCs) are often employed in bending-driven SBUV designs due to their low voltage requirements and inherent compatibility with aqueous media; dielectric elastomers (DEs) offer large strain capabilities and high energy density, facilitating rapid and forceful actuation; while hydraulically amplified self-healing elastomers (HASELs) combine electrically triggered deformation with hydraulic transmission, providing robust and scalable motion generation for biomimetic platforms. In this section, we review the working principles, representative SBUV implementations, and recent advances in these three prominent EAP-based actuation technologies. Figure 5 presents the schematic diagrams of the working principles of three types of electroactive polymers (EAPs), with detailed explanations provided in subsequent sections [129,130,131].

### 5.1. SBUVs Driven by Ionic Polymer–Metal Composite (IPMC) Soft Actuators

Ionic Polymer–Metal Composite (IPMC) is a typical electroactive polymer material characterized by low-voltage actuation, flexible deformation, and self-sensing capabilities [6]. Regarded as an “artificial muscle,” it shows considerable promise for applications in soft biomimetic underwater vehicles (SBUVs). A typical IPMC structure comprises a proton exchange membrane (e.g., Nafion) as the electrolyte layer, with noble metal electrodes (e.g., platinum or gold) deposited on both sides via electroless plating or sputtering [132]. Under a low direct current (DC) voltage (typically 1–7 V), hydrated cations (such as H^+^) within the membrane migrate toward the cathode. This ion flux carries water molecules, inducing an asymmetric swelling and contraction across the membrane thickness, which consequently results in bending deformation through an electro-chemo-mechanical coupling mechanism [6,132,133].

Beyond actuation, IPMC can generate electrical signals in response to mechanical deformation, enabling self-sensing. This dual functionality has attracted significant interest in biomimetic robotics, flexible sensing, and smart structures [134]. Its merits—including low driving voltage, minimal noise, light weight, softness, and good biocompatibility—make it well-suited for simulating flexible biological appendages such as fish fins and rays [135]. However, challenges such as limited output force, short operational lifetime in dry environments, and back-relaxation effects hinder its direct practical application [136].

To address these limitations, researchers have pursued improvements in material modification, electrode design, modeling, and control. Material-wise, incorporating polyvinyl alcohol (PEO), carbon nanotubes (CNTs), or graphene oxide (GO) can enhance ionic conductivity and water retention, improving environmental stability [137,138,139]. Electrode fabrication has also advanced; techniques such as porous metal structuring, electrochemical deposition, and magnetron sputtering improve electrode flexibility and interfacial adhesion [140,141,142]. Theoretically, beyond the classical Poisson–Nernst–Planck (PNP) model, nonlinear models incorporating thermo-mechanical coupling and reverse diffusion have been developed to better predict large deformations and back-relaxation [143,144,145]. In control, methods combining input shaping with self-sensing feedback have enhanced motion accuracy and system robustness [146,147].

In biomimetic fish propulsion, IPMC is often used to emulate the undulatory motions of pectoral and caudal fins, benefiting from its flexibility, ease of integration into streamlined bodies, and efficient low-voltage operation. By precisely regulating the input voltage, IPMC-based swimmers can replicate natural fish locomotion with lower energy consumption than conventional motor-driven systems. Noteworthy implementations include a serrated IPMC design by Chang et al. that improved propulsion efficiency [148], and g-C_3_N_4_ electrodes introduced by Wu et al. that enhanced high-frequency driving stability [149]. Furthermore, ionic liquid encapsulation has been shown to significantly extend IPMC service life in non-aqueous settings.

In summary, as a multifunctional smart material combining actuation and sensing, IPMC holds unique advantages for soft biomimetic robots, particularly in underwater propulsion. Future progress will likely rely on co-design across materials, structures, and control, along with interdisciplinary approaches to advance efficient, low-noise, and perceptive biomimetic fish systems.

IPMC has attracted sustained research interest in SBUVs due to its low-voltage operation, flexible deformation, fast response, and light weight. Since 2007, the field has evolved from initial prototypes to encompass theoretical modeling, performance enhancement, structural innovation, and intelligent control. In 2007, Ye et al. developed a centimeter-scale autonomous IPMC bionic robotic fish resembling a goldfish [150]. It integrated a microcontroller and infrared sensors for wireless control and obstacle avoidance. Measuring 98 mm × 30 mm × 22 mm and weighing 21.9 g, the prototype improved maneuverability by optimizing the caudal fin-to-flexible body ratio, setting a foundation for subsequent miniaturized and autonomous IPMC fish.

**Figure 6:**
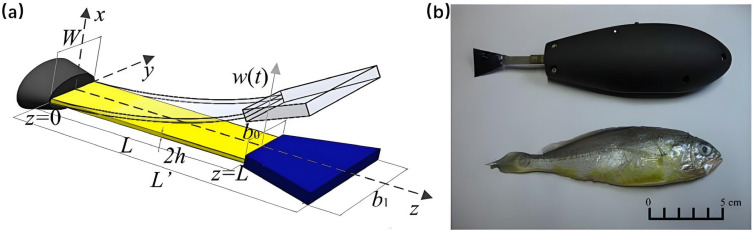
Example of a soft biomimetic underwater vehicle (SBUV) driven by an ionic polymer-metal composite (IPMC) actuator. (**a**) Modeling diagram of the IPMC caudal fin. (**b**) Robotic fish comprising a rigid body and an IPMC caudal fin [153].

In motion modeling, Aureli et al. (2010) established a dynamic model for an IPMC-based vibrating underwater vehicle, incorporating added mass and fluid damping effects with a modal reduction approach to identify thrust and torque [151]. This provided a theoretical basis for performance prediction and optimization. In 2013, Shen et al. built a hydrodynamic model using slender-body theory and evaluated thrust efficiency and self-propelled speed under various driving conditions via a servo-towing test system, deepening insight into IPMC propulsion mechanisms [152]. Their work, supported scale-effect analysis for IPMC SBUVs. In the same year, Shen et al. [153] applied analytical techniques and fuzzy logic methods to the dynamic modeling and efficient swimming control of a bionic robotic fish actuated by Ionic Polymer-Metal Composites (IPMC) (Figure 6). A physics-based bionic robotic fish model was proposed, which integrates the fluid mechanics of the IPMC tail and the actuation dynamics of the IPMC itself. Comparison between simulation and experimental results demonstrated the feasibility of the dynamic model. Using this model, they found that the harmonic control of the actuation frequency and voltage amplitude of the IPMC is the main mechanism for the robotic fish to achieve high thrust efficiency during swimming [153].

Building on these early model-based control foundations, recent research has intensified the focus on developing strategies that balance precision with computational efficiency for embedded systems. In 2024, Tomar et al. systematically compared three controllers—PID, fuzzy logic, and H∞ control—for regulating IPMC-based biomimetic thrusters, evaluating their accuracy, response speed, and noise rejection [154]. Advancing this in 2025, the same team adopted a more accurate fractional-order (FO) model of IPMCs and implemented both feedforward inverse model and PID control to precisely adjust bending displacement [155].

While such model-based approaches enhance precision, achieving stable, long-duration control of IPMCs’ highly nonlinear and hysteretic behavior remains challenging. To directly address the need for low-computational-load solutions, Chen et al. (2025) proposed a simplified scheme combining PWM driving with feedforward compensation, which does not rely on complex models [156]. This method achieved sustained underwater position control within ±4 mm for 8 min, reducing error by over 50% and demonstrating that robust closed-loop performance can be attained with minimal computational complexity [156].

Collectively, this evolution from early dynamic modeling to contemporary intelligent and simplified control schemes highlights a clear pathway toward efficient, low-computational-load control for IPMC-based swimmers, which is crucial for their practical deployment in autonomous SBUVs.

To improve the agility of SBUVs, Yang et al. (2015) introduced a multi-fin IPMC coordination strategy [157]. The robot used two IPMC pectoral fins for steering and one IPMC caudal fin for propulsion, reaching a forward speed of 0.5 cm/s and a turning rate of 1.5 rad/s, demonstrating good 2D maneuverability. In 2018, Chen et al. proposed a hybrid caudal fin combining a servo motor and IPMC [158]. The servo generated flapping propulsion while the IPMC provided steering, enabling a turning radius less than half the body length and significantly improving motion flexibility.

In 2021, advances continued in bio-inspired morphologies and intelligent control. Karelin et al. developed a stingray-like swimming robot [159] propelled by 12 IPMC actuators and two empennages, achieving speeds up to 40 mm/s. Also that year, Yi et al. implemented a coordinated collision-avoidance strategy for a two-joint caudal fin robotic fish [160]. The approach integrated data-driven modeling with the collision cone concept, improving adaptability in complex environments.

In structural optimization, Ji et al. (2023) designed an IPMC-based bionic pectoral fin inspired by biological fin mechanics [161]. Using four fins with force compensation, the robot achieved stable straight-line swimming at 1 mm/s, offering benefits in structural simplicity, low noise, and high controllability.

In summary, over more than a decade of development, IPMC-driven SBUVs have achieved substantial progress in structure design, modelin, and control. Future work focusing on improving actuation efficiency, environmental durability and integrating intelligent sensing and autonomy will further promote their application in underwater exploration and monitoring.

### 5.2. SBUVs Driven by Dielectric Elastomer Actuators (DEAs)

Dielectric elastomer (DE) is an emerging electroactive polymer with a typical sandwich structure: a flexible dielectric membrane sandwiched between two compliant electrodes. Its actuation relies on Maxwell stress induced by an applied high voltage (typically in the kilovolt range), which squeezes the membrane, resulting in large in-plane expansion. Due to its large strain, high energy density, and fast response, soft actuators based on DE is well-suited to drive SBUVs [7].

Electrode materials critically influence DEA performance. Conventional options include conductive grease, carbon spray, graphite powder, and liquid electrolytes. Studies show that electrode conductivity, compliance, and compatibility with the elastomer substrate significantly affect actuation strain and efficiency [162]. Recently, nanomaterials such as carbon nanotubes (CNTs) have been widely adopted, offering high conductivity and mechanical flexibility, which improve linear strain, actuation efficiency, and operating voltage [163,164,165]. Furthermore, electrode fabrication methods—including screen printing, inkjet printing, and metal sputtering—also influence breakdown strength and environmental robustness, with rigid metal electrodes generally offering higher breakdown fields than carbon-based ones [166].

Elastomer selection is equally important. Silicone elastomers are widely used for their fast response and cycling stability. Composite modifications—such as incorporating barium titanate (BaTiO_3_) or conductive polyaniline—can raise the dielectric constant and tailor the elastic modulus, enabling larger deformation at lower voltages [167,168,169]. Advanced processing techniques like micro-patterning and additive manufacturing (3D printing) further support precision fabrication and performance tuning of DEA-based devices [170].

For practical underwater deployment, the long-term stability and fatigue resistance of DEAs in submerged environments remain critical challenges, addressed through two main material-level strategies. The first strategy involves external medium modification to improve electrical reliability. La et al. (2013) demonstrated that submerging DEAs in dielectric silicone oil quenches electrical breakdown, allowing operation at electric fields over 800 MV/m (1.8 times higher than in air) and achieving 140% areal strain, thereby suppressing a key failure mode in aqueous environments [171]. The second strategy focuses on developing intrinsic self-healing systems to combat mechanical fatigue directly. Liu et al. (2024) developed a self-healing DEA with a compatible electrode, achieving 50.6% driving strain [172]. When integrated into a self-powered system, it recovered over 90% of its performance after 10 damage-healing cycles, demonstrating potential for long-term durability under cyclic loading. Together, these approaches of external protection and internal self-repair provide complementary pathways toward solving the reliability concerns in aqueous operation [172].

Beyond the challenges specific to aqueous environments, other reliability concerns also limit service life and performance. These include interlayer mechanical mismatch and poor adhesion, which constrain displacement output [173]. Additionally, electrode architecture itself is crucial; for large-strain applications, multi-layer CNT-based electrode structures help homogenize the electric field and mitigate capacitance loss due to pinholes, thereby maintaining high performance [174].

Dynamic modeling of DEs continues to be an active research area. Nonlinear material models (e.g., Neo-Hookean, Yeoh) combined with electromechanical coupling theories have been used to analyze electrically induced deformation, instability, and material nonlinearity [175,176], providing a basis for design optimization.

Dielectric elastomer actuators (DEAs), with their muscle-like properties, have become an important platform for SBUVs. With ongoing advances in materials and fabrication, DEAs have achieved remarkable improvements in efficiency, response speed, operating voltage, and longevity, which enable their diverse applications. Research in this domain has progressed from basic validation to high-performance, multifunctional, and intelligent systems. The following prototypes highlight this evolution.

Early studies focused on underwater feasibility. In 2016, Shintake et al. introduced a laminated silicone insulation structure, their fish-like and jellyfish-like robots reached speeds of ~8 mm/s and 1.5 mm/s, respectively, at 3 kV [177]. The same year, Godaba et al. demonstrated a DEA-driven jellyfish robot with fast response and good load capacity [178], while Henke’s team realized a fully soft, rigid-free robot, suggesting new paths to DEA autonomy [179].

**Figure 7:**
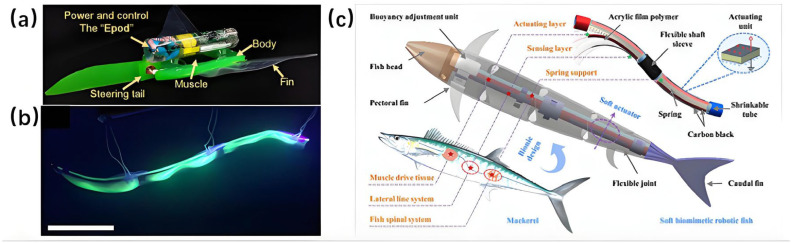
Examples of SBUVs driven by dielectric elastomer actuators (DEAs). (**a**) Fish robot driven by a dielectric elastomer and ionically conductive hydrogel [180]. (**b**) Translucent soft robots driven by frameless fluid-electrode DEAs [182]. (**c**) Soft robotic fish with embedded sensing for adaptive multi-mode swimming [185].

Subsequent efforts emphasized structural optimization and environmental adaptability. In 2017, Li et al. proposed a conductive fluid electrode design [180], as shown in Figure 7a. By using grounded fluid as the electrode, the robot reached 9.2 mm/s in saltwater and addressed buoyancy control. In 2021, the same team developed an untethered soft robotic fish for deep-sea operation [181], successfully activating it at 10,900 m depth in the Mariana Trench, confirming DEA functionality under extreme pressure.

Since 2018, DEA structures have trended toward frameless and miniaturized designs. Christianson et al. introduced a frameless configuration where the actuating electric field is established by using the internal fluid as one electrode and the surrounding environmental liquid as the other, both serving as the necessary conductive pathways [182], as shown in Figure 7b. Their transparent eel-larva robot swam at 1.9 mm/s with 52% Froude efficiency and 94% optical transparency. Also in 2018, Berlinger et al. created a micro autonomous underwater vehicle with modular DEA fins, enabling untethered motion without pre-stretch and improving system integration [183]. Shintake et al. presented the design and characterization of a DEA-based BCF soft robotic fish [184].

Bio-inspiration sources have also diversified. In 2019, Wang et al. built a dual-DEA robotic frog inspired by breaststroke [186]. Weighing 14.3 g, the robot exhibited multi-mode mobility. In 2022, Jiao et al. designed an antagonistic DEA and applied it to a BCF robotic fish [185], as shown in Figure 7c, achieving a maximum speed of 22.7 mm/s. In 2023, Wang et al. designed a DEA manta ray that reached 58 mm/s with a 26° flapping angle under 4 kV and 4 Hz drive [187]. In 2025, He et al. used a swing linkage to amplify DEA output for large-angle pectoral fin motion. Their study showed that traveling wave undulation enhanced speed, thrust, and efficiency [188]. The same year, He et al. developed a bioinspired soft swim bladder (BSSB) [189], achieving a 4.2 mL volume change under 10 kV and opening a path for oarless propulsion.

Recently, DEA systems have moved toward intelligent integration. In 2024, Yamaguchi et al. developed a tapered DEA module mimicking fish white muscle [190]. It produced 7.4° bending and 2.06 N thrust at 10 kV and was used in a multi-module robotic fish. In 2025, Wang et al. realized an “actuation–sensing–control” integrated soft robotic fish [191] that adaptively switches swimming modes via multi-unit coordination and environmental perception, marking a key step toward DEA intelligence.

Despite clear advantages, DEAs still face challenges including high operating voltage, material fatigue and limited durability. Future advances in materials, structural design, and system integration will be crucial to deploying them in complex underwater environments.

### 5.3. SBUVs Driven by Hydraulically Amplified Self-Healing Electrostatic (HASEL) Actuators

Hydraulically Amplified Self-Healing Electrostatic (HASEL) actuators were first introduced by Prof. Christoph Keplinger’s group at the University of Colorado Boulder. They operate by using electrostatic forces to redistribute a liquid dielectric within a flexible shell, producing muscle-like contraction. Key innovations include self-healing capability—where the liquid dielectric recovers insulating properties after electrical breakdown—and high power density, with strain rates exceeding 900%/s [192,193]. A basic HASEL consists of a flexible shell (e.g., silicone or BOPP film), compliant electrodes, and a liquid dielectric. Applied voltage generates Maxwell stress, displacing the liquid and deforming the shell. By varying electrode geometry, output force and strain can be tuned, leveraging hydraulic amplification [194].

Research has focused on improving performance through materials, drive electronics, and control. Early work emphasized geometry and material modeling. Kellaris et al. developed an analytical model for Peano-HASEL actuators, increasing energy density via parameter optimization [195]; Mitchell et al. created a rapid prototyping toolkit, enabling >100% linear strain at >100 Hz and accelerating high-performance HASEL development [196]. Electrically, Lodh et al. designed an ultra-high-gain converter that boosts 5–10 V inputs to 5 kV outputs, easing the integration of high-voltage drives into compact systems [197,198]. Gravert et al. used high-k dielectrics to lower operating voltages below 1 kV, improving portability and safety [199].

In sensing and control, Vogt et al. proposed the F-HASEL architecture, integrating dedicated sensing electrodes for real-time displacement monitoring [200]. Xiong et al. introduced elastic elements with AC excitation (SE-HASEL), reducing hysteresis and output decay [201]. Cisneros et al. applied the Port-Hamiltonian framework and IDA-PBC control to improve robustness in curled HASEL actuators [202]. Structurally, Tynan’s sliding mechanism and Hu’s rigid electrode design improved coupling efficiency and output performance, respectively [203,204].

For system integration, Johnson et al. combined HASEL actuators with circuits and magnetic feedback in a 10 × 10 soft skin array, showing potential for human–machine interaction and haptics [205]. Currently, HASELs are used in soft robots, wearables, and haptic systems, though challenges remain in operating voltage, durability, and multi-actuator control. Continued co-development of materials, electronics, and control will expand their practical use.

In summary, electrohydraulic soft actuators have recently achieved substantial gains in voltage reduction, motion performance, and system integration. Future materials and control advances should further their role in marine exploration and environmental monitoring.

Recent advances in HASEL soft actuators (2023–2025) have markedly improved the performance of underwater soft robots, with clear trends toward high efficiency, low voltage and functional integration.

**Figure 8:**
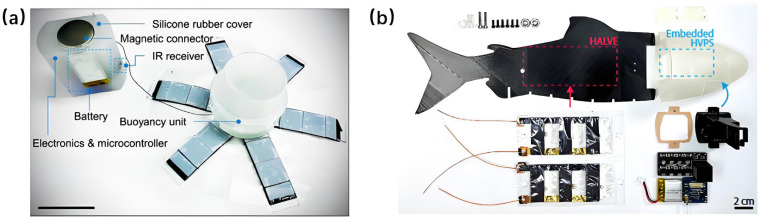
Examples of SBUVs driven by hydraulically amplified self-healing electrostatic (HASEL) actuators. (**a**) A versatile jellyfish-like robotic platform [207]. (**b**) Low-voltage electrohydraulic actuators for untethered robotics [199].

In 2023, Shibuya et al. proposed a silicone-insulated HASEL actuator design, achieving bending and linear modes via single- or double-sided insulation [206]. A 20 mm × 40 mm bending actuator attained 39.0° bending and 9.6 mN blocking force at 10 kV, while a linear version achieved 956.1% contraction strain at 6.6 kV, showing promise for biomimetic underwater robots. Also in 2023, Wang et al. developed a multi-functional jellyfish-like robot [207] (Figure 8a) combining HASEL actuators with a rigid-soft hybrid structure. It achieved 6.1 cm/s upward propulsion with ~100 mW input, plus object stabilization, fluid mixing, and multi-robot coordination, highlighting hybrid design benefits for functional integration.

In 2024, Gravert et al. optimized HASEL materials and structure, reducing drive voltage to 1100 V (a 4.9× reduction) while maintaining high power density (50.5 W/kg) and strain rate (971%/s) comparable to mammalian muscle [199]. As shown in Figure 8b, their soft biomimetic fish used a BoPVDF shell and dielectric oil filling for improved underwater reliability. Also in 2024, Zhang et al. created a manta ray-inspired robot using electrohydraulic soft actuators [208]. An improved electrode encapsulation method allowed an underwater flapping angle of 68°, near aerial performance. The robot reached 111.2 mm/s (2.22 BL/s) cruising speed and 96°/s turning rate, demonstrating high agility and low-noise propulsion.

In 2025, Hartmann et al. demonstrated a centimeter-scale high-performance soft robot [209] mimicking marine flatworms. It used traveling-wave pectoral fins driven by monolithically integrated electrohydraulic actuators with a PVDF-terpolymer dielectric, operating below 500 V. The robot swam at 12 cm/s tethered and 5.1 cm/s untethered, combining high speed and autonomy in a miniaturized platform. While HASEL actuators exhibit impressive muscle-like strain rates, their deployment in underwater environments introduces distinct multi-physics constraints, as rightly noted by the reviewer. External hydrostatic loading can pre-compress the actuator shell, elevating the electrostatic force required for initiation and altering force–strain dynamics. Concurrently, the coupling between rapid electromechanical response and the viscous damping of the surrounding fluid may limit effective bandwidth and energy transfer efficiency. Although dedicated studies on perfectly compensating for these effects in HASELs are still emerging, recent research trends provide clear pathways to address these challenges without fundamentally compromising compact design. Innovations in material systems and geometric design (e.g., using high-permittivity dielectrics to lower voltage [210,211]) collectively aim to enhance intrinsic performance against environmental pressures. A prime example is the development of high-strain Peano-HASEL actuators, where novel materials and geometries have achieved muscle-like strains exceeding 24%. This intrinsic enhancement of fundamental kinematic performance provides a greater performance margin to counteract potential losses induced by the aquatic environment. In parallel, integrated sensing and control strategies, such as embedding capacitive sensing electrodes for real-time feedback [200], enable intelligent compensation within a highly compact form factor. Furthermore, co-design approaches leverage analytical models for actuator optimization [195] to holistically improve performance margins against damping losses and pressure effects. Therefore, the prevailing strategy to overcome underwater limitations hinges not on add-on mechanisms but on the continued, synergistic co-development of materials, fabrication, and embedded intelligence, ensuring that future HASEL-based SBUVs remain high-performing, agile, and compact.

While IPMC, DEA and HASEL actuators all enable biomimetic underwater propulsion, their operational principles and performance profiles differ substantially, presenting a trade-off space for SBUV design. IPMC actuators offer the advantages of low-voltage operation (1–7 V) and inherent self-sensing in aqueous environments, but are limited by low output force and back-relaxation. DEAs excel with large strain, high energy density, and fast response, yet typically require high kilovolt-scale driving voltages and face challenges regarding dielectric reliability. HASEL actuators combine electrostatic driving with hydraulic amplification to achieve high power density and a unique self-healing capability against electrical failure, though they also currently operate at high voltages and their system integration can be complex. A concise comparison of these key characteristics is provided in Table 2, highlighting their respective strengths and challenges for SBUV applications.

## 6. Existing Challenges and Future Trends

Recent years have witnessed continuous advancements in the actuation technologies for SBUVs. Different actuation methods exhibit distinct characteristics in terms of performance and application suitability. Pneumatic systems, for instance, are valued for their simple structure, fast response, and capacity for large-amplitude motion, yet their relatively large volume (causing buoyancy interference) and dependence on an external air supply limit their use in miniaturized platforms and long-duration autonomous missions. Hydraulic soft actuators enable flexible and stable motion with high efficiency, but they often involve bulky components and high energy consumption. Dielectric Elastomer Actuators (DEAs) offer high energy density and excellent compliance, facilitating lifelike continuous deformation; however, they require high-voltage operation, and their service life is significantly constrained by material aging. Electrohydraulic actuation combines the high output force of hydraulic systems with the control convenience of electric drives, though challenges remain in system complexity, sealing reliability, and long-term durability. Ionic Polymer–Metal Composites (IPMCs) can produce substantial bending deformation under low voltage, are lightweight, and perform well in humid conditions, but their practical application is hindered by limited output force, inadequate durability, and sensitivity to environmental humidity. Shape Memory Alloy (SMA) based soft actuators feature a compact structure, high power density, and ease of achieving large deformations, but they are generally hampered by slow response speeds, low energy efficiency, and finite fatigue life.

In summary, the prevailing research trend in SBUV propulsion is shifting toward lightweight, integrated, and high-efficiency solutions, with increasing emphasis on stability and durability in complex underwater environments. It is unlikely that any single actuation method can simultaneously fulfill all demands for high speed, long endurance, and precise control. Future progress in SBUVs is therefore expected to increasingly rely on hybrid actuation strategies that combine multiple technologies. The example of Li et al. (2022), who integrated dielectric elastomers (DEs) and twisted and coiled polymer fibers (TCPFs) into a dual-mode structure, provides a conceptual blueprint for SBUVs [212]. Translating this to aquatic propulsion, a promising approach could involve the co-deployment of actuators with complementary characteristics—for instance, pairing high-speed, low-force HASELs for rapid body undulation with high-force, slower hydraulic actuators for powerful caudal fin beats or attitude adjustment. Their synchronization to achieve unified locomotion would hinge on a layered control architecture: a high-level bio-inspired central pattern generator (CPG) to dictate the overall gait and timing, coupled with low-level feedback loops utilizing integrated self-sensing (e.g., from F-HASELs) to precisely regulate the phase, amplitude, and force output of each actuator type in real-time. To ensure the durability and long-term stability of such advanced prototypes across diverse marine conditions, establishing standardized benchmarking is crucial. This entails the development of modular testing platforms for fair comparison, graded environmental testing from laboratory to field conditions, and embedded sensing for long-term performance monitoring, collectively forming a “design-test-data-optimization” closed loop. Within this loop, high-fidelity fluid–structure interaction (FSI) modeling and simulation will emerge as a pivotal enabling technology. As emphasized in Section 2.2, FSI establishes a mandatory co-design paradigm for SBUVs. Advancing this paradigm, future high-fidelity FSI simulations will allow for the virtual exploration of how soft body deformation, actuation patterns, and hydrodynamic performance interrelate in complex flow fields. This capability will enable the optimization of the synergy between actuation strategy, material mechanics, and morphology during the design phase, significantly accelerating prototype iteration by reducing reliance on costly and time-intensive trial-and-error testing in physical tanks. Together, the development of such smarter material systems, deeply integrated control architectures, rigorous benchmarking methodologies, and enabling tools like FSI simulation, will ultimately propel the evolution of biomimetic SBUVs toward unprecedented levels of fidelity, adaptability, and versatility in complex underwater missions.

## Figures and Tables

**Table 1:** Summary of key soft material classes for biomimetic underwater robots.

Material Class	Typical Examples	Elastic Modulus	Density (g/cm^3^)	Actuation Compatibility & Key Traits	Primary Advantages	Main Limitations
Silicones	Ecoflex, PDMS, Dragon Skin	10^4^~10^6^ Pa (Very low)	0.9~1.2	Fluid-powered (Pneumatic/Hydraulic): Excellent. Large strain, fast response. Chemically inert.	High elasticity, ease of molding and bonding, transparency, biocompatibility	Low tensile strength, prone to tear, high gas permeability
Hydrogels	PVA, Alginate, PEG-based	10^3^~10^6^ Pa (Tunable, low)	1.0~1.2	Swelling/Electrochemical: Stimuli-responsive (pH, ion). Self-healing possible. Slow actuation.	High water content, biocompatibility, adhesive, stimuli-responsive	Mechanically weak, prone to dehydration/swelling, slow dynamics
Electroactive Polymers (EAPs)						
Dielectric Elastomers (DEs)	Acrylic (VHB)	10^6^~10^7^ Pa (Low)	0.9~1.1	Electrostatic (High Voltage): Very high strain and energy density, fast response.	Large strain, high energy density, fast response	Requires kV-level voltage, dielectric breakdown, viscoelastic losses
Ionic Polymer- Metal Composites (IPMCs)	Nafion with Pt/Au electrodes	10^8^~10^9^ Pa (Medium)	1.5~2.5	Ion Migration (Low Voltage < 5 V): Bending mode, inherent self-sensing, silent actuation	Low-voltage, biomimetic bending, sensing capability	Small force output, relaxation under DC, dry out or hydrolyze
Shape Memory Alloys (SMAs)	NiTi (Nitinol)	30~80 GPa (High)	6.4~6.5	Thermal (Joule Heating): High force, large recovery stress. Cyclic frequency limited by cooling	High force-to-weight ratio, compact size	Low efficiency (≤5%), fatigue, hysteresis, slow cycle time
Smart Material Composites	Elastomer matrix with fibers/particles	Tunable (Wide Range)	Varies	Defined by Matrix & Filler: Combines matrix compliance with functional filler response.	Enhanced properties (strength, conductivity); multi-functional	Fabrication complexity, potential inter-facial failure

**Table 2:** Performance comparison of SBUVs based on three types of EAP actuators.

Propulsion Mechanism	Dimension (mm)	Speed (mm/s; BL/s)	Driving Voltage (V)	Performance Advantages and Metrics	Source
SBUVs Based on IPMC Actuators					
BCF + MPF	length: 230	5; /	<2	XBee wireless control; turning speed of 1.5 rad/s; low consumption; no motor gears, quiet and stealthy; suitable for underwater sensor networks.	[157]
BCF	length: 270 width: 80	120; /	7.3	Turning speed of 40 deg/s; turning radius < 0.5 body lengths; complete 2D dynamic model; balanced thrust and flexibility.	[158]
MPF	/	40; /	4	Autonomous WiFi remote control (ESP-12E module); smartphone control via MQTT server; multiple IPMCs connected in parallel to enhance thrust.	[159]
BCF	/	/	2.8~4.85	Dual-robot cooperative collision avoidance (collision cone method); salinity affects the relaxation effect; data-driven modeling; smooth steering.	[160]
MPF	40 × 18 × 12	1; /	5	Supports three gaits: forward movement, in-place turning, and arc turning; excellent trajectory stability; no noise and low power consumption.	[161]
SBUVs based on DE actuators					
MPF	length: 93	135; 1.45	10,000	Endurance 3 h 15 min; temperature tolerance 0.4–74.2 °C; transparent stealth.	[180]
BCF	length: 220 height: 50	1.9; 0.009	7500	Transmittance 94%; fluorescent communication; acoustic stealth (0.3 dB increase).	[182]
MPF	100 × 30 × 60	55; 0.55	2000	Endurance > 100 min; turning radius 120 mm (1.2 BL); supports planar swimming + vertical diving.	[183]
BCF	length: 150	37.2; 0.25	5000	Strouhal number 0.47 (close to real fish); body length 150 mm.	[184]
MPF	100 × 123 × 66	76.7; 0.77	4800	Turning radius 70 mm; self-adaptive foot structure (speed increased by 3.15 times).	[185]
BCF	175 × 50 × 70	22.7; 0.14	3000–7000	Maximum output force 48.54 mN; bilateral antagonistic actuation.	[186]
BCF	/	20.38; /	5000	First-order natural frequency in air 4.05 Hz; performance improved after streamlined optimization.	[187]
MPF	240 × 119 × 15	36; /	5000	Turning radius 185 mm; angular velocity 8.5°/s; optimal performance when fin thickness is 0.2 mm.	[188]
Multi-BCF	160 × 53 × 56	45; 0.28	6000	4 swimming modes; adaptive to environmental viscosity sensing; no significant biological disturbance.	[191]
SBUVs based on HASEL actuators					
Jellyfish	diameter: 160	61; 0.38	6500	Contactless object manipulation; fluid mixing; steering; wireless swimming; multi-robot collaboration; shape adaptation.	[207]
BCF	length: 28	38; 0.14	1100	Underwater propulsion; self-recovery (withstands dielectric breakdown).	[199]
MPF	length: 50 width: 50	111.2; 2.22	8000	Fast linear propulsion; agile steering (96.4°/s, turning radius 11.13 cm); freshwater/saltwater compatible.	[208]
MPF	45 × 55 × 0.5	120; 2.6	<500	Surface propulsion; agile steering (195°/s); can push objects 16 times its own weight; navigates through narrow spaces/grass.	[209]

## Data Availability

No new data were created or analyzed in this study.
